# Ankle Kinematics Characterization in Children with Idiopathic Toe Walking: Does the Foot Model Change the Clinical Evaluation?

**DOI:** 10.3390/healthcare11060873

**Published:** 2023-03-16

**Authors:** Paolo Brasiliano, Martina Alvini, Eugenio Di Stanislao, Giuseppe Vannozzi, Giuseppe Di Rosa, Valentina Camomilla

**Affiliations:** 1 Department of Movement, Human and Health Sciences, University of Rome “Foro Italico”, Piazza Lauro De Bosis 6, 00135 Rome, Italy; p.brasiliano@studenti.uniroma4.it (P.B.); valentina.camomilla@uniroma4.it (V.C.); 2 Interuniversity Centre of Bioengineering of the Human Neuromusculoskeletal System, University of Rome “Foro Italico”, 00135 Rome, Italy; 3 “ITOP SpA Officine Ortopediche”, Via Prenestina Nuova 307/A-Area Industriale, 00036 Palestrina, Italy; 4 Division of Pediatric Neurorehabilitation, “Ospedale Pediatrico Bambino Gesù”, 00165 Rome, Italy

**Keywords:** ITW, multi-segment foot model, pediatric gait, gait analysis, rockers

## Abstract

Idiopathic toe walking (ITW) is a gait deviation characterized by forefoot contact with the ground, sometimes observed in children, that alters ankle kinematics, possibly leading to health-related issues. When studying foot and ankle gait deviations, the adoption of a single-segment foot model entails a significant simplification of foot and ankle movement, and thus may potentially mask some important foot dynamics. Differences in ankle kinematics between single- (conventional gait model, PiG, or Davis) and multi-segment (Oxford foot model, OFM) foot models were investigated in children with ITW. Fourteen participants were enrolled in the study and underwent instrumented gait analysis. Children were asked to walk barefoot and while wearing a foot orthosis that modified the ankle movement pattern toward a more physiological one without blocking foot intrinsic motion. ITW gait abnormalities, e.g., the absence of heel rocker and the presence of anticipated forefoot rocker, were found/not found according to the foot model. Walking conditions significantly interacted with the foot model effect. Finally, the different characterization of gait abnormalities led to a different classification of ITW, with a possible impact on the clinical evaluation. Due to its closer adhesion to ankle anatomy and to its sensitivity to ITW peculiarities, OFM may be preferable for instrumented gait analysis in this population.

## 1. Introduction

Toe walking is a gait deviation characterized by forefoot ground contact and by marked ankle plantarflexion over the entire gait cycle. It is a gait pattern that is present in several pathologies, such as autism spectrum disorders, cerebral palsy, muscular dystrophy, and others [1]. At the same time, it is commonly seen temporarily during typical gait development [2]. In some cases, typically developed children who should have developed a physiological heel-to-toe gait may present a toe-walking gait pattern. If a clinical cause cannot be identified, they are diagnosed with idiopathic toe walking (ITW) [1]. Persistent ITW has been hypothesized to lead to limitation in ankle range of motion (ROM) together with issues such as higher risk of falling or psychological discomfort [3,4,5]. A recent review provided an overview of the available methods used to quantify changes of gait pattern in children with ITW [6]. In 63% of the studies included in the review, parameters obtained through instrumented gait analysis were used as primary outcomes of the investigation. The characterization of the differences compared to normal gait is an essential step to obtain a deep understanding of the pathology; in this context, instrumented gait analysis is mandatory to assess the kinetics and kinematics of the foot and ankle joints. Indeed, the main gait deviations caused by this condition occur at the ankle joint, altering its kinematics [7]. Ankle joint function is usually described in terms of angular excursions and foot rockers: the heel, ankle, and forefoot rocker [8]. Together with the analysis of ankle sagittal moment, the characterization of these rockers has been used to determine the severity of ITW [9]. The severity classification of ITW has been used previously to monitor treatment effects [6]. Moreover, it could be integrated within the clinical evaluation of children with ITW performed by physicians, allowing objective assessment of the status of the condition.

Ankle kinematics is commonly determined using stereophotogrammetry, modeling the foot as one rigid segment (as in the conventional gait models plug-in gait (PiG) [10] and Davis (DAV) [11]). However, in the past years, multi-segment foot models (MSFMs) have been widely used, especially in clinical populations [12]. MSFMs are collectively considered to describe the anatomy of the foot more accurately. When the anatomical description of a body segment changes, the estimation of the adjacent joint kinematics and kinetics changes. In the case of the foot, changing its anatomical description (mono- or -multi-segmented foot) will change the characterization of the ankle and the foot intrinsic joint movement. MSFMs have been found to be effective in distinguishing between normal and pathological feet [12]. Even if the evidence is still preliminary, other uses of the MSFMs have been found in pathological populations: surgery outcome assessment, correlation of foot pathologies with proximal joint movement deviation or other symptoms, association of foot pathologies with patients’ reported outcomes, classification of foot types, and MSFM repeatability assessment [12]. Among MSFMs, the Oxford foot model (OFM) has been validated [13] and is commonly used in children [12]. In some cases, MSFMs lead to different characterizations of ankle kinematics at critical points of the gait cycle [14]. Nevertheless, even if the differences in ankle kinematics estimation between mono- and multi-segment foot models have been assessed, their impact on the clinical evaluation of ITW still needs to be verified.

To date, scientific literature has not analyzed the impact of the foot model selection on the resulting ankle kinematics and clinical evaluation in children with ITW. It is noteworthy that in the case of toe walking, the assumption of a rigid and non-deformable foot (i.e., as when using a mono-segment foot model) ascribes the movement of the foot intrinsic joints to the ankle joint, possibly modifying its functional assessment. While MSFMs can more accurately describe the anatomy of the ankle joint and change the estimation of its kinematics, they pose some issues. The motion-capture system resolution must be sufficient to track a high number of adjacent markers, especially when assessing the gait of children with small feet. In addition, the operators must be familiar with the palpation of additional anatomical landmarks compared to the standard mono-segment foot model used in clinical gait analysis. Lastly, the participants may experience the experimental setup as less comfortable (due to a longer preparation time and higher number of markers necessary to record walking trials using MSFM). Given these considerations, before choosing an MSFM over a mono-segment foot model to perform instrumented gait analysis, proof of relevance for a clinical evaluation when using the former over the latter must be obtained.

As a first aim, this study evaluated the effects of using mono- or multi-segment foot models to analyze ankle kinematics in children with ITW. The interest was to verify whether the use of a biomechanical model that better describes the anatomy of the ankle leads to different results in terms of severity classification of ITW as support to the clinical evaluation. To this end, instrumented gait analysis was performed using OFM (version 4 and 5 described in the work by Stebbins and colleagues [13]), PiG, and DAV models on children with ITW walking barefoot. As a second aim, the study verified whether the differences between models are specific to the presence of ITW gait deviations (i.e., verifying whether a model × walking condition interaction exists). For this purpose, children were also asked to walk while wearing a foot orthosis that promoted heel-to-toe gait [15], changing the ITW typical gait pattern towards a more physiological one.

## 2. Materials and Methods

Fourteen children diagnosed with ITW were enrolled in the study (8 females, age = 9 ± 2 years, mass = 37 ± 15 kg, stature = 1.38 ± 0.14 m) following signed consent by their parents. The study received the approval of the local institutional review board (University of Rome “Foro Italico”, Rome, Italy, CAR130/2022). Children were diagnosed by a physician of the “Bambino Gesù” hospital in Rome and referred to ITOP “Officine Ortopediche” for screening assessment comprising clinical evaluation with instrumented gait analysis.

**Figure 1:**
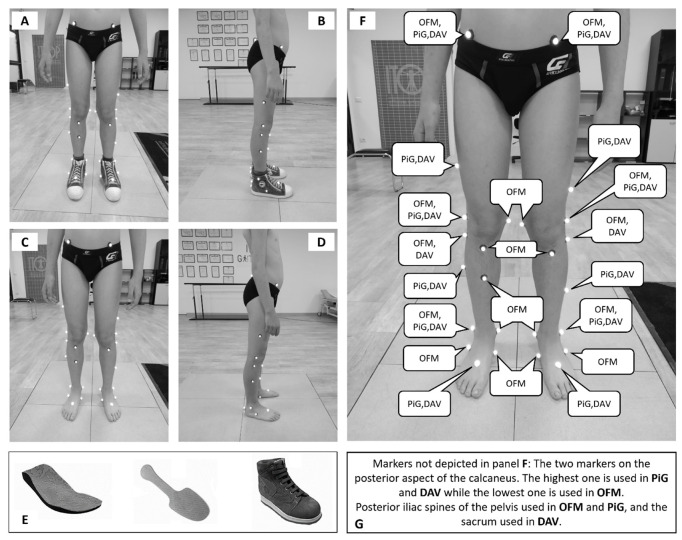
Front and lateral view of the used marker set while wearing the foot orthosis (**A** and **B**, respectively) and in barefoot condition (**C** and **D**, respectively). Bottom (**E**): representation of the A. Dyn.O.^®^ orthosis. From left to right: foot orthosis, carbon fiber flexible plate, orthopedic shoe. (**F**) Graphical representation of markers used in the different models, with specification (**G**) for the markers not depicted in panel (**F**). Relevant coordinate systems are described in the Supplementary Materials.

During testing sessions, children were asked to walk barefoot (BF) and while wearing a foot orthosis (FO) at their preferred speed along a straight 8 m walkway. A motion-capture system with eight infrared cameras (BTS SMART-DX, Quincy, USA, @250 frame/s) and four floor-embedded force plates (BTS Bioengineering Corp, Quincy, USA, @1000 frame/s) were used to measure gait kinematics and to detect gait events and define gait cycles, respectively. A total of 113 and 115 gait cycles were used for BF and FO conditions, respectively, corresponding to a mean of about 6 complete gait cycles per subject (range 4 to 10). Participants started walking with their preferred leg. A trial was considered valid when a complete foot contact on at least one force platform was obtained. Markers were placed on the child’s lower limbs to allow ankle kinematics reconstruction according to the OFM, PiG, and DAV (Figure 1A–D) models, and kinematic data were obtained (Nexus 2.10, Vicon, Oxford, UK). Raw data were filtered using a low-pass fourth-order Butterworth filter (fc = 12 Hz). The orthosis (A.Dyn.O.^®^, ITOP SpA “Officine Ortopediche”, Palestrina (RM), Italy) used is a modular solution that combines a custom-made insole, a carbon fiber flexible plate and a specific orthopedic shoe (Figure 1E). The orthosis is designed to exert a downward force on the hindfoot, with the aim of contrasting the toe-walking pattern without blocking the ankle. More precisely, when the carbon fiber plate is solicited by an external force in the anterior portion (i.e., in the case of a forefoot contact with ground), it stores elastic energy that is successively expressed in the posterior region of the plate. This produces a dorsiflexor moment at the ankle joint, dragging down the hindfoot. The textile design of the shoe used with the orthosis allowed for palpation of all the anatomical landmarks of the models except for the posterior aspect of the calcaneus (CA), which was selected as the posterior edge of the orthosis along the line of the Achilles tendon. In the mono-segment foot models, CA height with respect to the marker placed on the base of the metatarsal head was the only factor influencing sagittal kinematics estimation. The shoe used with the orthosis did not hinder the correct relative positioning of these markers. In the multi-segment foot model, CA was used together with the other markers placed on the calcaneus to determine the sagittal plane of the hindfoot. As far as its position is at the same height of the medial and lateral markers placed on the calcaneus and along the line of the Achilles tendon, it did not affect ankle sagittal kinematics estimation. In addition, the orthosis was firmly fixed to the foot, minimizing its in-shoe movement.

**Figure 2:**
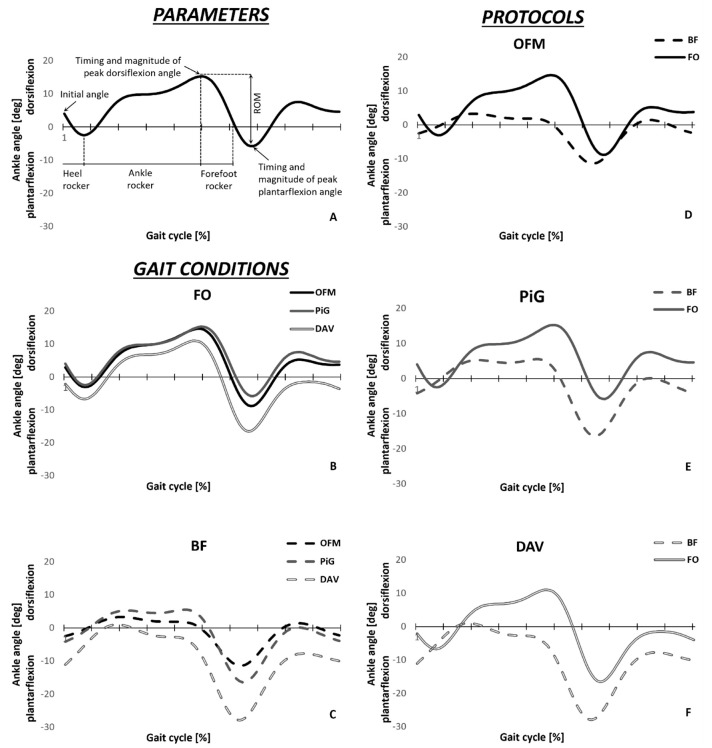
(**A**): Graphical representation of calculated parameters on a representative kinematic trace obtained using OFM. In all other panels the mean ankle kinematics of the participants’ representative trials estimated using OFM (black), PiG (dark gray), and DAV (dark gray contoured) for FO (solid) and BF (dashed) conditions is represented. In (**B**,**C**), curves from different models are compared for FO (solid) and BF (dashed) conditions, respectively. In (**D**–**F**), curves from different conditions are compared for OFM (black), PiG (dark gray), and DAV models, respectively.

For each subject and each model, a representative gait cycle was selected looking at ankle planta-dorsiflexion angle using the method proposed by Sangeux and colleagues [16]. The method calculates the area between each kinematics trace of a given set of waveforms and a median waveform calculated as the median value of the set at each time instant. Preliminary to the statistical analysis, inter-participant consistency was verified for each model using the linear fit method (LFM) [17]. LFM gives information about the strength of the relationship (R^2^) between the subjects’ kinematic traces. An R^2^ greater than 0.5 was found for the two conditions, and thus inter-subject consistency was proved. Timing and magnitude of peak plantar and dorsiflexion angles, along with ankle angle at foot contact and plantar-dorsiflexion ROM were measured (Figure 2A) for the subjects’ representative gait cycles.

To verify whether the main effects of using mono- or multi-segment foot models existed and whether the differences between models were specific to the presence of ITW gait deviations, i.e., whether interactions between models and walking conditions existed, a 3 × 2 repeated measure ANOVA was performed (SPSS 23.0, Chicago, IL, USA) on the above-mentioned parameters. In case of lack of normality assumption, the non-parametric factorial model was performed using the ARTool R package [18]. Effect size was assessed through η2 values. Significance level was set at 0.05 for all statistical tests, using Bonferroni correction for post-hoc comparisons.

Additionally, for all gait cycles of the BF condition, the presence/absence of a heel rocker and a premature forefoot rocker were investigated as key kinematic features following the most common ITW severity classification [9]. According to this classification, a first rocker is identified with an ankle angle at foot contact higher than −5 deg with a down-going pattern angular excursion within the first 12% of the gait cycle. An early third rocker is defined as the maximal ankle dorsiflexion angle occurring before the 30% of the gait cycle.

## 3. Results

Both the interaction term (upper part) and main effects for models and walking conditions (lower part) are reported in Table 1. The main effects for walking condition and interaction, speculating only on the FO condition, are not commented on, as they do not relate to the aims of this work.

Concerning the post-hoc comparisons for the walking conditions, a difference in ankle ROM between FO (25.3 ± 3.6 deg) and BF (16.6 ± 4.8 deg) walking was detected only using OFM (Figure 2D). The timing of the forefoot rocker was found to be significantly different between walking conditions only when using OFM and DAV models (Figure 2D–F). Differences between conditions in peak plantarflexion angle were found using PiG and DAV models (Figure 2E,F).

Concerning the post-hoc pairwise comparisons for the model type in BF condition (Figure 2C), ankle ROM was found to be significantly different across all models, with OFM (16.7 ± 4.8 deg) showing the smallest value, followed by PiG (26.3 ± 6.3 deg) and DAV (31.4 ± 5.1 deg). The timing of the forefoot rocker was found to be different when using PiG (35 ± 14% of gait cycle) compared with the DAV (23 ± 8% of gait cycle), with the former being delayed. Lastly, peak plantarflexion angle was found to be significantly different across all models, with OFM (−12.4 ± 5.2 deg) showing the smallest value, followed by PiG (−17.4 ± 6.4 deg) and DAV (−28.7 ± 6.3 deg).

Concerning the effect of foot models, regardless of the walking condition, DAV model underestimated the ankle angle at foot contact (−7.3 ± 8 deg) compared to OFM (−0.5 ± 7.6 deg) and PiG (1 ± 6.8 deg). It also underestimated the magnitude of peak dorsiflexion angle during the stance phase compared to PiG (6.9 ± 8.2 deg and 12.2 ± 5.6 deg, respectively, Figure 2B,C).

**Figure 3:**
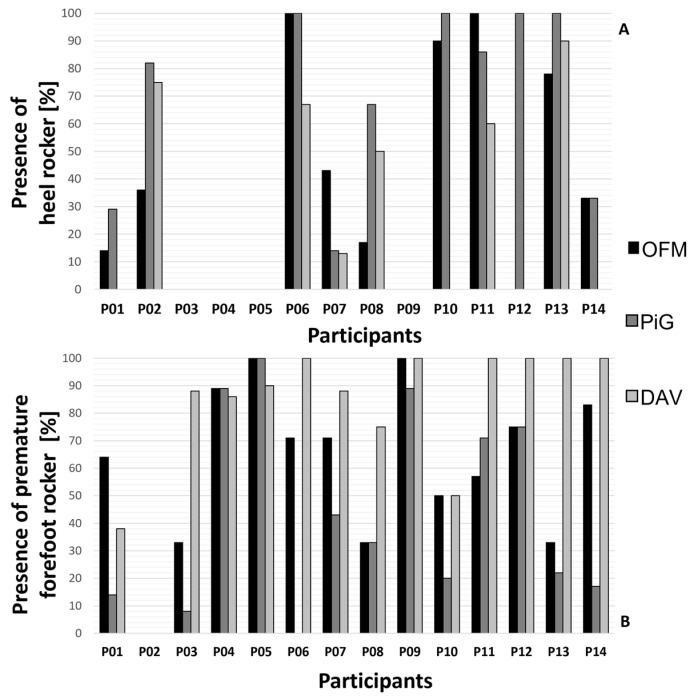
Graphical representation of the percentage of the gait cycles in which: (**A**) a heel rocker (up) and (**B**) a premature forefoot rocker (down) were detected using OFM (black boxes), PiG (dark gray boxes), and DAV (light gray boxes) for each participant.

Participants’ characterization of heel rocker and premature forefoot rocker are shown in Figure 3A,B. The heel rocker was detected in 35%, 44%, and 26% of all recorded gait cycles using OFM, PiG, and DAV, respectively. A premature forefoot rocker was detected in 61%, 40%, and 79% of all recorded gait cycles, using OFM, PiG, and DAV, respectively.

## 4. Discussion

The present work evaluated the role of mono- and multi-segment foot models in describing ankle kinematics as a support to the clinical evaluation and classification of children with ITW walking barefoot and when wearing a foot orthosis.

The characterization of the ITW typical gait deviations changed according to the adopted foot model. As shown by the post-hoc comparisons for the model type, differences in ankle ROM between OFM and PiG were found in BF conditions only, while peak plantarflexion angle difference between OFM and PiG changed its sign in the BF and FO conditions. Moreover, differences in the timing of peak dorsiflexion angle between PiG and DAV were found in BF conditions only. In addition, comparison of walking conditions led to different results according to the model used for ROM, time of peak dorsiflexion, and peak plantarflexion angle. Therefore, the choice of the model changed the characterization of the kinematics parameters used for ITW severity classification (i.e., heel and forefoot rockers, Figure 3A,B). 

The significative interaction effects found in this study demonstrate that the choice of biomechanical model is a critical factor when comparing different gait patterns. Indeed, when using a mono-segment foot model to estimate ankle kinematics, the motion of the intrinsic foot joints is ascribed to the ankle joint only. This may be mainly due to the use of a marker on the forefoot to analyze the motion of the hindfoot. Considering that all the models rely on the posterior aspect of the calcaneus to define the primary axis of the foot (or the hindfoot), the differences in the orientation of this axis are related to the second marker used to define it. The OFM uses a virtual marker that belongs to the calcaneus, while PiG and DAV use a marker on the forefoot. This may not largely alter the orientation of the anatomical coordinate system used to define the foot (or the hindfoot), in orthostatic position. Nevertheless, when the foot moves, the axis orientation changes to a greater degree, as it is related to two different bones that are not directly linked one to the other. This causes the ascription of the movement of the foot intrinsic joints to the ankle joint (for a detailed description of the anatomical coordinate systems definition for each model see Supplementary Materials).

Using a less detailed anatomical description of the foot causes alterations in ankle kinematics estimation specific to children with ITW. Figure 2A,B graphically shows how the differences in ankle kinematics estimation may affect the classification of ITW, and potentially the clinical evaluation performed using instrumented gait analysis. Nevertheless, it must be considered that the classification proposed was designed using ankle kinematics and kinetics derived from mono-segment foot model. The inconsistency in the characterization of the heel and forefoot rockers between mono- and multi-segment foot models highlights how a different kinematic estimation may change the clinical evaluation of ITW. Nevertheless, to minimize the differences related to the use of the foot model (rather than to the change in gait pattern), the classification proposed should be used to compare two groups, or the same group in different conditions. 

The choice of the biomechanical model may change the clinical evaluation of ITW as well as the assessment of the efficacy of treatments. Indeed, foot models performed differently both when considering barefoot conditions only and when assessing differences between walking conditions (e.g., when assessing the efficacy of treatments).

The results presented must be interpreted in the light of the limitation that only ankle kinematics has been evaluated. Nevertheless, it has been demonstrated that this joint is the one mainly affected by this gait deviation [7]. In addition, only the sagittal component of ankle kinematics was analyzed, since the literature still reports issues about the estimation of the other two ankle kinematics components [19,20]. Moreover, it is the ankle kinematics component that is mainly analyzed in this population. Lastly, even if the use of a foot orthosis tends to restore normal gait patterns in children with ITW, comparison with a group of typically developing children may give additional insights into the effects of the foot model on the clinical evaluation of this population.

To conclude, differences in ankle kinematics estimation between mono- and multi-segment foot models specific to this population have been found. Such discrepancies lead to different classifications of relevant features of this condition, thus changing the clinical evaluation performed using instrumented gait analysis. When feasible, the use of a multi-segment foot model is preferable when analyzing ankle kinematics, due to its more accurate anatomical description of the ankle joint.

## Figures and Tables

**Table 1:** Summary of statistical results. In the case of model × walking interaction (first part of the table), post-hoc comparisons are detailed for walking condition and model separately. When interaction was not significant (second part of the table), only main effects were reported for walking condition and model separately. RoM: range of motion; FO: foot orthosis condition; BF: barefoot condition; OFM: Oxford foot model; PiG: plug-in gait; DAV: Davis.

	Interaction Term (Model × Walking Condition)	Interaction Post-Hoc Comparisons for Walking Condition	Interaction Post-Hoc Comparisons for Model Type
Ankle RoM	F(2,26) = 29.976 (*p* < 0.05) η2 = 0.833	OFM_BF_ < OFM_FO_ (*p* < 0.05)	OFM_FO_ < DAV_FO_ PiG_FO_ < DAV_FO_ (*p* < 0.017) OFM_BF_ < DAV_BF_ < PiG_BF_ (*p* < 0.017)
Time of peak dorsiflexion angle (forefoot rocker)	F(2,26) = 4.459 (*p* < 0.05) η2 = 0.103	OFM_BF_ < OFM_FO_ DAV_BF_ < DAV_FO_ (*p* < 0.05)	PiG_BF_ > DAV_BF_ (*p* < 0.017)
Ankle peak plantarflexion angle	F(2,26) = 10.390, (*p* < 0.05) η2 = 0.444	PiG_FO_ < PiG_BF_ DAV_FO_ < DAV_BF_ (*p* < 0.05)	DAV_FO_ < PiG_FO_ < OFM_FO_ (*p* < 0.017) DAV_BF_ < OFM_BF_ < PiG_BF_ (*p* < 0.017)
	**Interaction term** **(model x walking condition)**	**Main effect forwalking condition**	**Main effect and post hoc for model type**
Time of peak plantarflexion angle	*p* > 0.05	F(1,13) = 192.012 (*p* < 0.05) η2 = 0.811 BF < FO (*p* < 0.05)	NS
Ankle angle at foot contact	*p* > 0.05	F(1,13) = 20.265 (*p* < 0.05) η2 = 0.609 BF < FO (*p* < 0.05)	F(2,26) = 27.980 (*p* < 0.05) η2 = 0.683 DAV < OFM, DAV < PiG (*p* < 0.017)
Ankle peak dorsiflexion angle	*p* > 0.05	F(1,13) = 99.203 (*p* < 0.05) η2 = 0.884 BF < FO (*p* < 0.05)	F(2,26) = 7.912 (*p* < 0.05) η2 = 0.378 DAV < PiG (*p* < 0.017)

## Data Availability

The data presented in this study are available on request from the corresponding author. The data are not publicly available due to privacy restrictions.

## Supplementary Materials

The following supporting information can be downloaded at: https://www.mdpi.com/article/10.3390/healthcare11060873/s1, File S1: Segments anatomical coordinate systems definition [21,22].
