# Supplementary material for: Ankle Kinematics Characterization in Children with Idiopathic Toe Walking: Does the Foot Model Change the Clinical Evaluation?
Source: Healthcare (Basel). 2023 Mar 16;11(6):873. doi: 10.3390/healthcare11060873 (PMC10047957; doi:10.3390/healthcare11060873)
Supplement: Supplementary file 1 [file healthcare-11-00873-s001.zip › healthcare-2164484-supplementary.pdf]

Segments anatomical coordinate systems definition

This document gives a brief description of the
segment definition for the three models used in the article.
Moreover, it gives a plausible explanation of the source of the differences
between the models found in the article.

Plug-in
Gait
Model

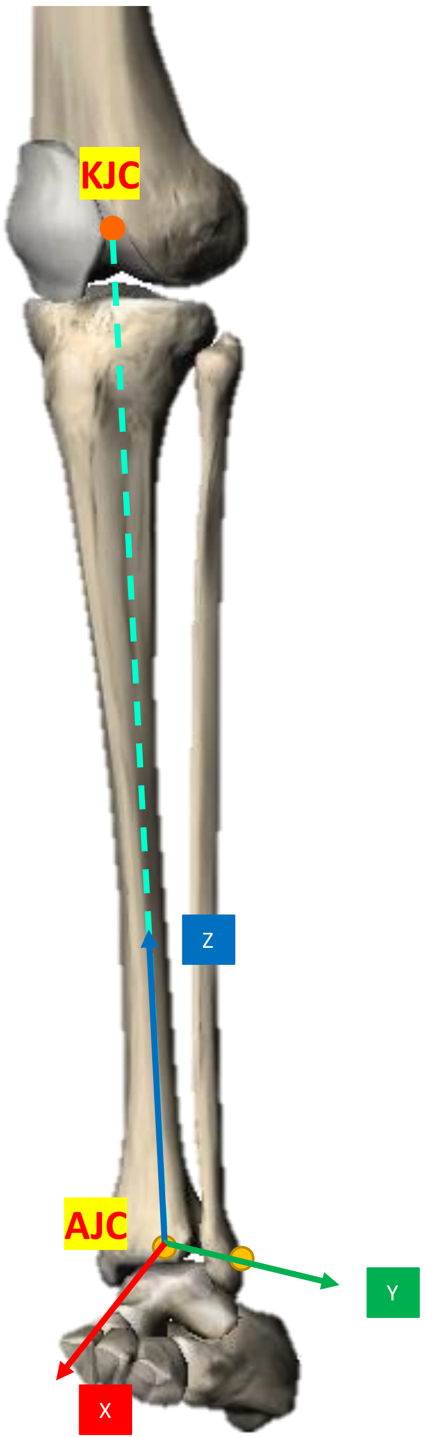

SHANK
Tibia is defined with an origin at the ankle joint center (AJC), the Z Axis in the direction from the AJC to the knee joint center (KJC), the Y axis leftwards along the line between the AJC and ANK marker, and the X axis generally forwards. This is representative of the distal end of the tibia.
[From Vicon Plug-in Gait reference guide, available at https://docs.vicon.com/display/Nexus214]

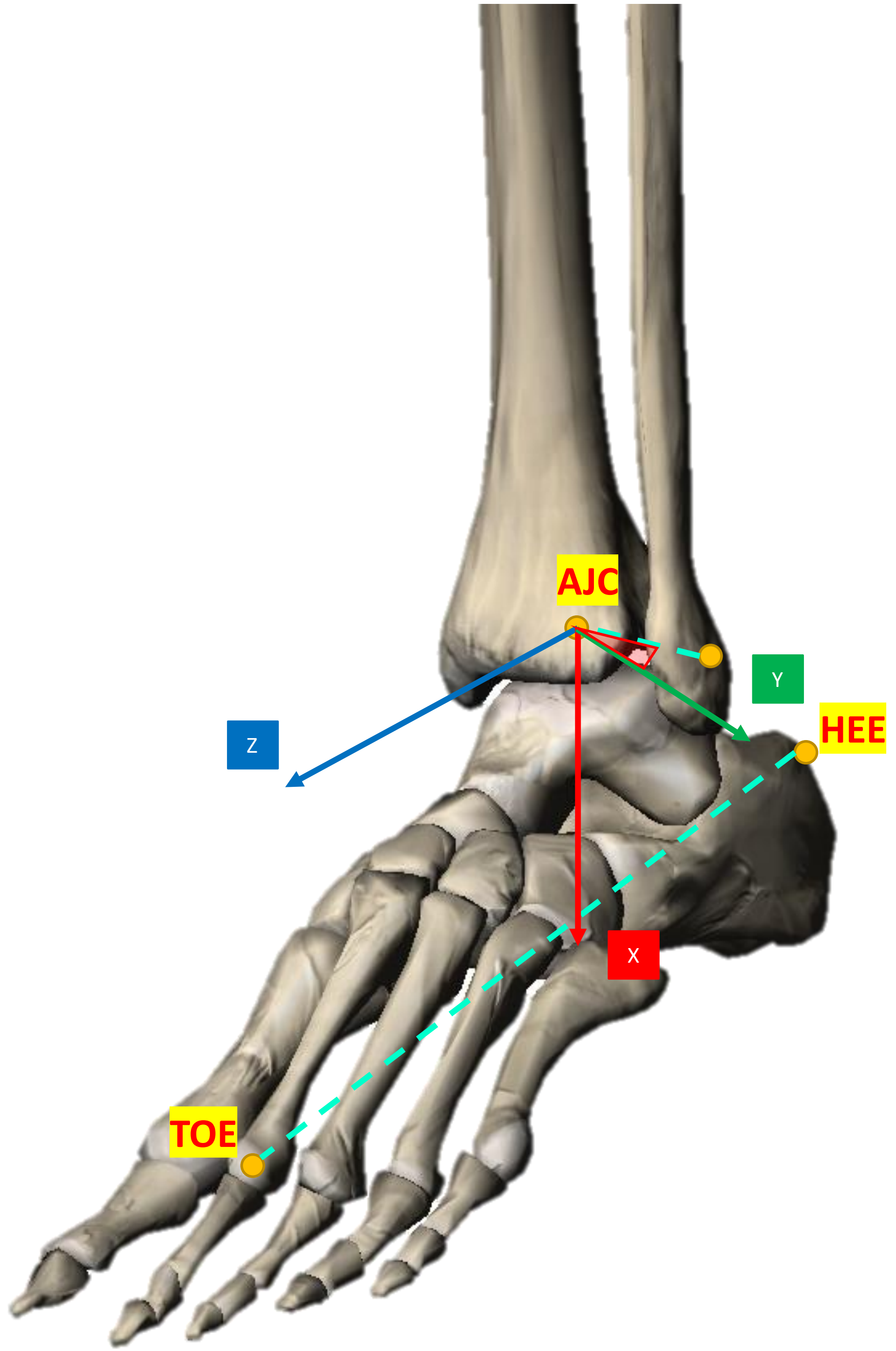

STATIC FOOT

The main foot segment is constructed using the TOE-HEE line as the primary axis. This line is taken as the Z axis, running forwards along the length of the foot. The direction of the Y axis from the tibia is used to define the secondary Y axis. The X axis thus points down, and the Y axis to the left.

A second foot segment is constructed, using the TOE-AJC as the primary axis, and again the Y axis of the untortioned tibia to define the perpendicular X axis and the foot Y axis (the “uncorrected” foot).

The Static offset angles (Plantar Flexion offset and Rotation offset) are then calculated from the 'YXZ' Cardan Angles between the two segments (rotating from the 'uncorrected' segment to the heel marker-based foot segment). This calculation is performed for each frame in the static trial, and the mean angles calculated. The static plantar-flexion offset is taken from the rotation around the Y axis, and the rotation offset is the angle around the X axis. The angle round the Z axis is ignored.

[From Vicon Plug-in Gait reference guide, available at https://docs.vicon.com/display/Nexus214]

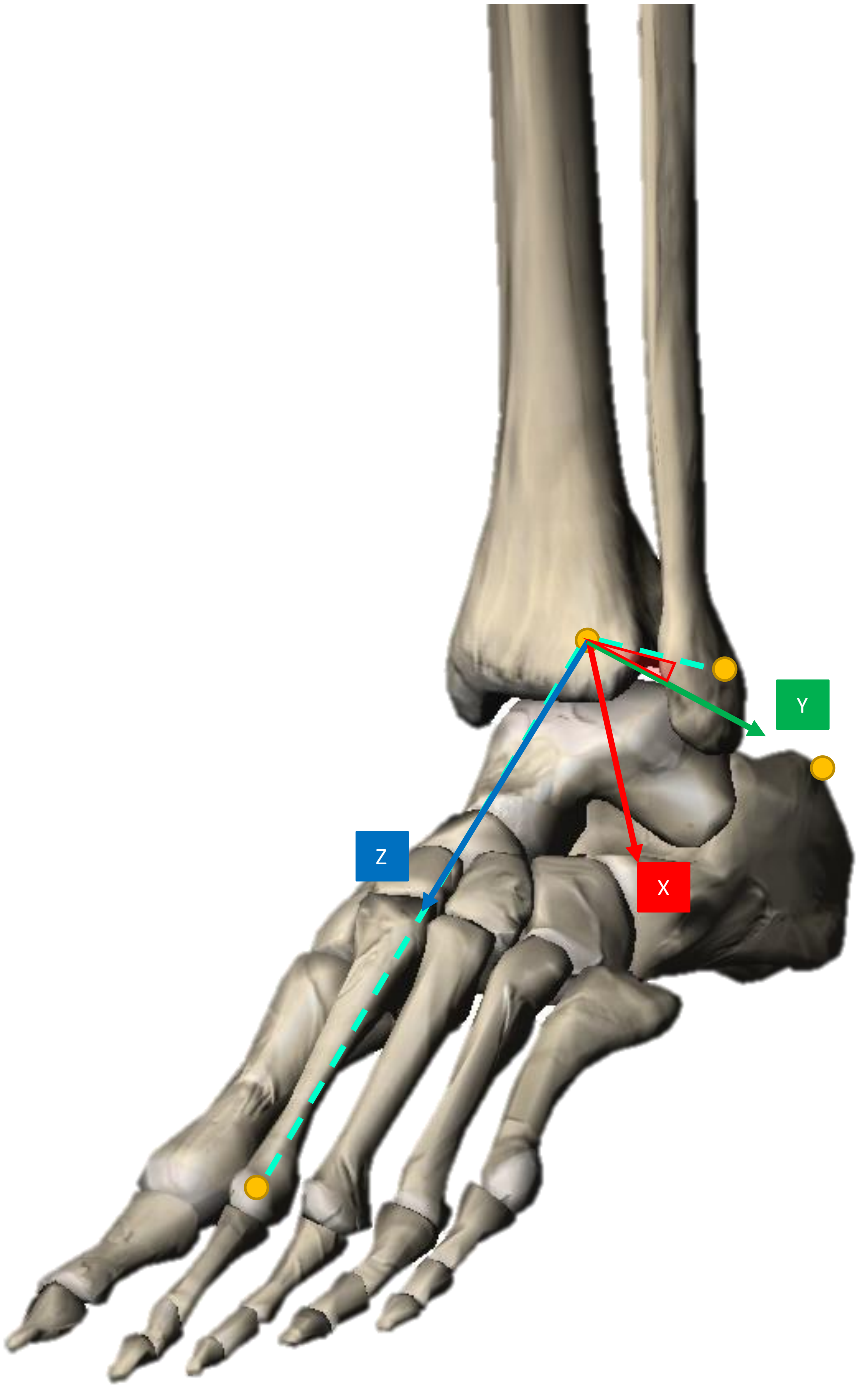

DYNAMIC FOOT

In the dynamic trial, the foot is calculated in the same way as for the “uncorrected” foot. The resulting segment is then rotated first round the Y axis by the Plantar Flexion offset. Then the resulting segment is rotated around its X axis by the rotation offset.

[From Vicon Plug-in Gait reference guide, available at https://docs.vicon.com/display/Nexus214]

Conventional Gait Model

Given that the kinematic model for DAV is not freely available,
It will be assumed to be similar to the Conventional Gait Model.
Nevertheless, some discrepancies may be present.

[The Conventional Gait Model - Success and Limitations, in Handbook of Human Motion, Richard Baker et al.]

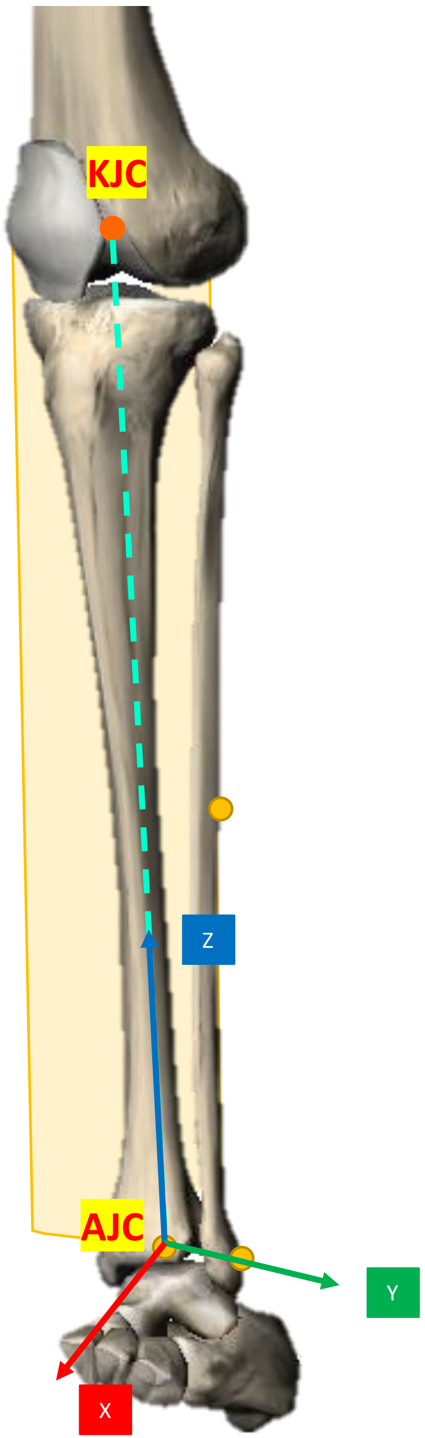

SHANK

The vertical axis is defined going from the AJC to the KJC. The shank frontal plane is defined by the AJC, the KJC and the marker on the lateral side of the shank. The anterior posterior axis is defined perpendicular to this plane, pointing forward. The mediolateral axis is defined as the cross product of the vertical and anterior-posterior axes.

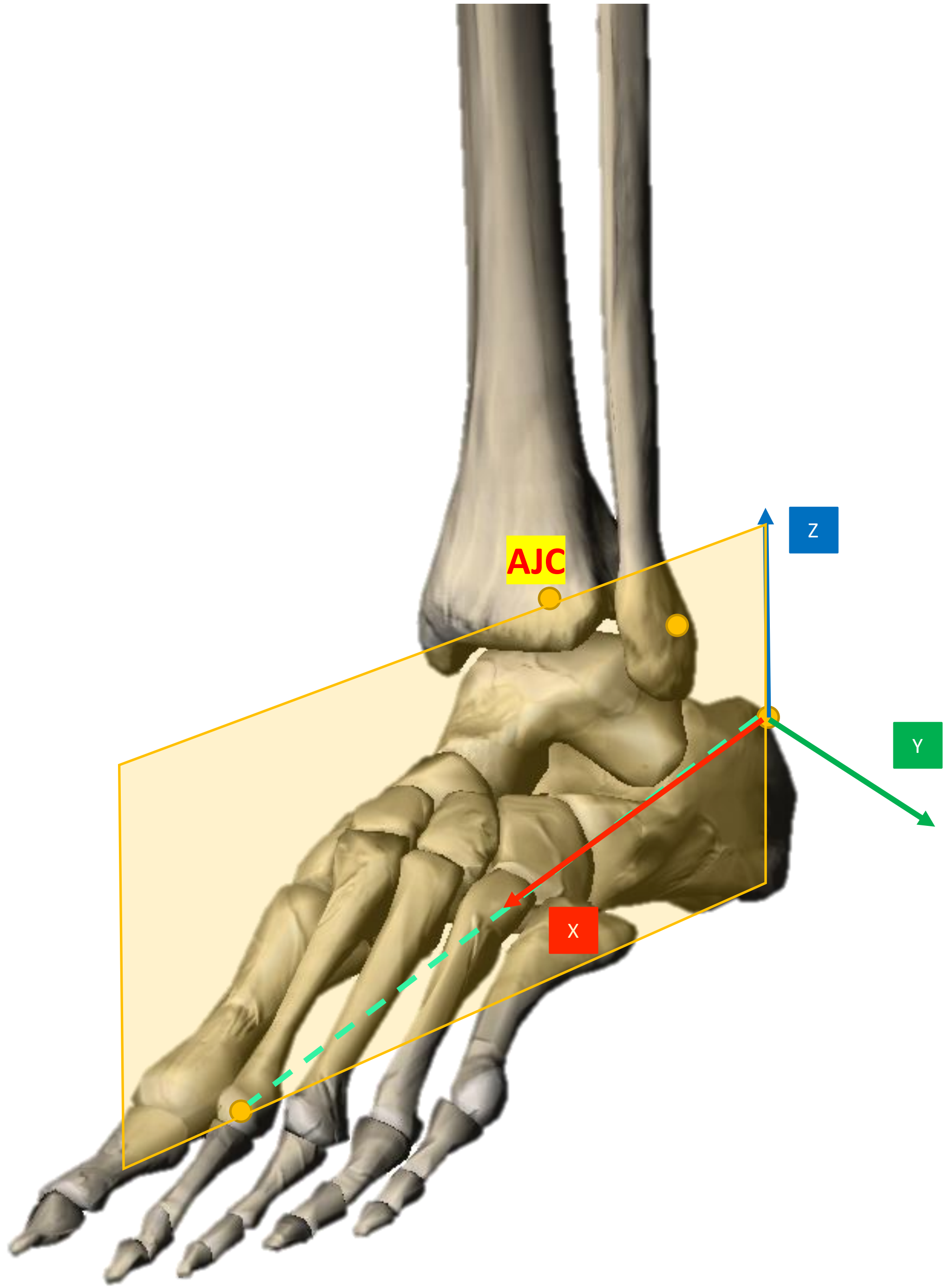

FOOT

In the original work [Davis et al., 1991], the foot is described as a vector rather than using an orthogonal coordinate system. Two vectors were defined during the static trial, one going from the posterior aspect of the calcaneus to the base of the second metatarsal head, the other going from the AJC to the base of the second metatarsal head. The angle offset between the two vectors is calculated on the sagittal and horizontal planes. During dynamic trials, the foot was represented by the vector going from the ankle joint center to the base of the second metatarsal head rotated by the offset angles. Nevertheless, when using a 3D coordinate system to represent the foot it may be done as in PiG or as follow:

The primary axis is defined as going from the posterior aspect of the calcaneus to the base of the second metatarsal head. The foot sagittal plane is defined by the two above mentioned points and the ankle joint center. the mediolateral axis is perpendicular to this plane, and the vertical axis is the cross product of the first two axes.

Oxford Foot Model

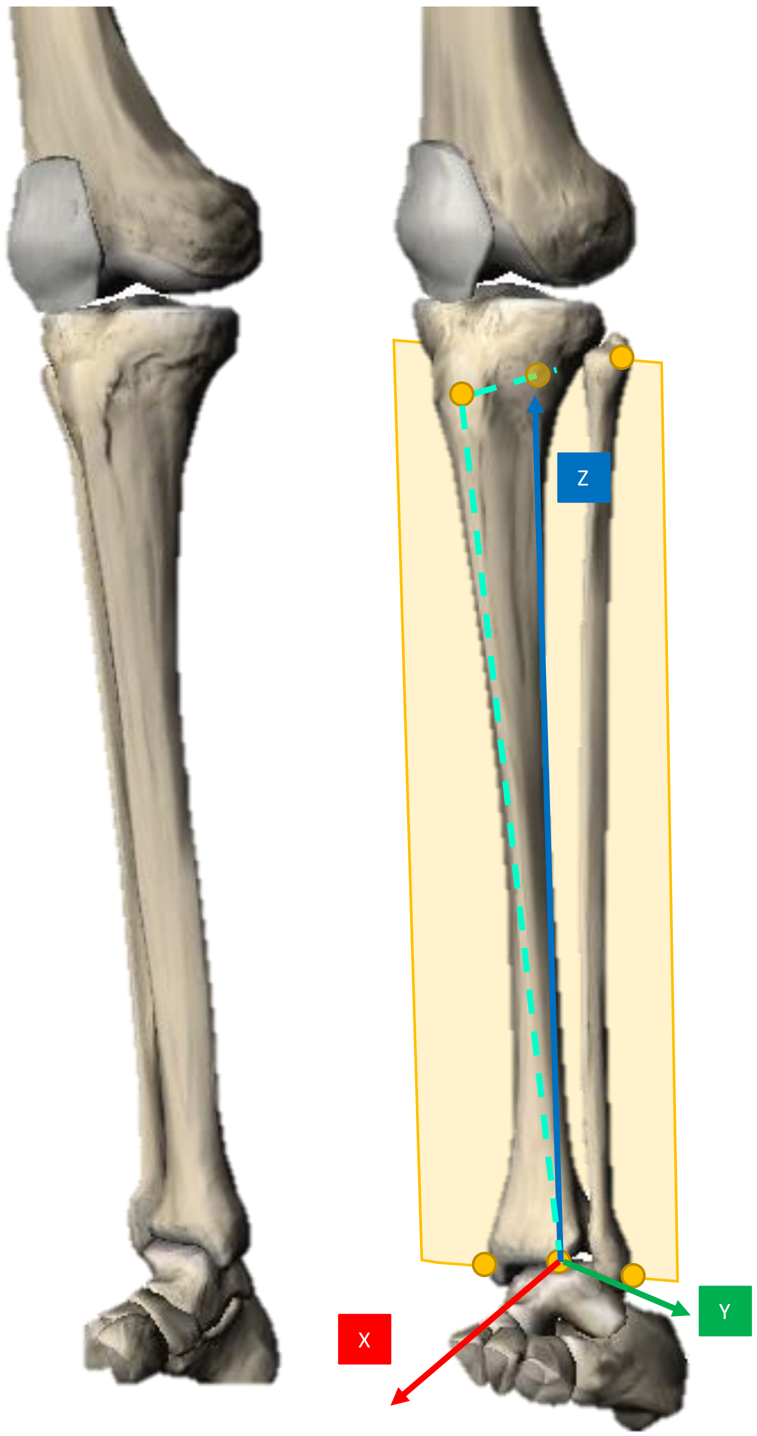

SHANK
First the shank frontal plane is defined using the markers on the malleoli and the marker on the head of the fibula. The vertical axis is defined going from the malleolar mid-point to the projection of the marker placed on the tibial tubersity on the shank frontal plane. The anterior posterior axis is defined perpendicular to this plane, pointing forward. The mediolateral axis is defined as the cross product of the vertical (Y) and anterior-posterior (X) axes.

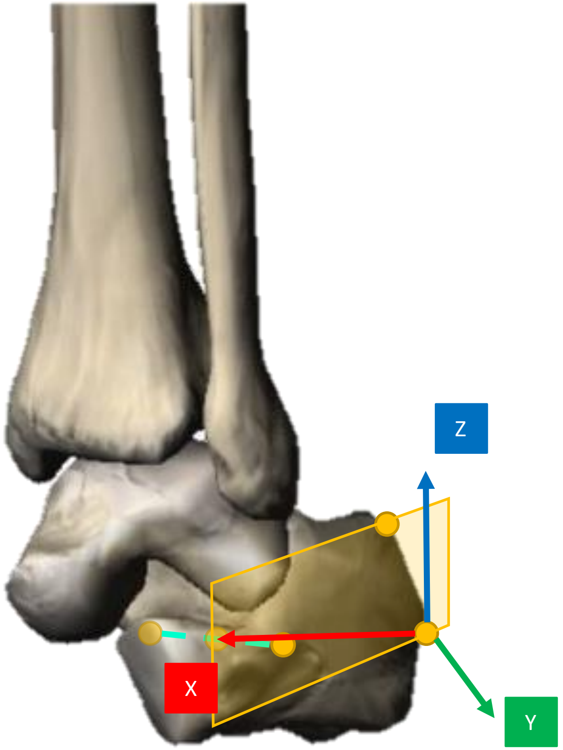

HINDFOOT

First the hindfoot sagittal plane is defined using the markers on the sustentaculm talii, the lateral aspect of the calcaneus and the posterior aspect pf the calcaneus. The anterior-posterior axis is defined to be lying on this plane and to be parallel to the floor. The mediolateral axis is defined perpendicular to this plane. The vertical axis is defined as the cross product of the mediolateral (Y) and anterior-posterior (X) axes.

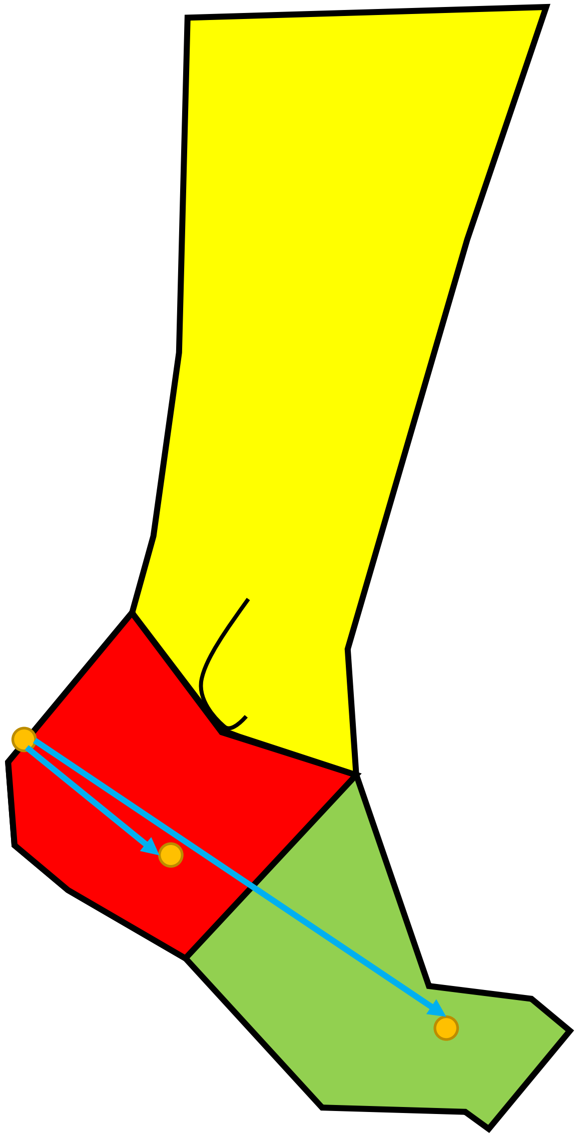

Apart from the differences in the anatomical coordinate systems definitions (both for the shank and the foot), it is plausible that the main source of discrepancies between mono and multi-segment foot model is the use of markers placed on different bones of the foot. In the case of the mono-segment foot models, the use of the marker on the second metatarsal head will cause a different orientation estimation of the foot compared to the orientation obtained using markers placed on the calcaneus only.
